# Supporting Staff in Southern Family Planning Clinics: Challenges and Opportunities

**DOI:** 10.1007/s10995-021-03339-5

**Published:** 2022-01-08

**Authors:** Anna Newton-Levinson, Megan Higdon, Roger Rochat

**Affiliations:** 1 Hubert Department of Global Health, Rollins School of Public Health, Emory University, 1518 Clifton Road, Atlanta, GA 30322 USA; 2 Department of Behavioral Sciences and Health Education, Rollins School of Public Health, Emory University, 1518 Clifton Road, Atlanta, GA 30322 USA

**Keywords:** Workforce, Access, Family planning, Abortion, Clinic staff

## Abstract

**Objectives:**

The aim of this study was to identify key challenges and opportunities to better support non-clinician clinic staff at family planning centers in Southern US states.

**Methods:**

We conducted qualitative interviews with 15 individuals in clinic staff and leadership positions at family planning centers in seven Southern states.

**Results:**

Turnover had negative impacts on both clinic functioning as well as patient care. Participants identified several challenges related to recruitment and retention in family planning health centers in the South, including the conservative contextual landscape, the perceived value of support staff, gaps in communication, and rural locations. In response to these challenges, staff also identified key strategies to better support and retain health center workers. These included prioritizing investment in management, creating career advancement opportunities, prioritizing staff retention, and creating space for self-care. Health center staff and leadership who used these strategies to support and retain staff noted improvements in the effectiveness of staff work as well as increases in patient volume.

**Conclusions for Practice:**

Study findings provide key areas for intervention including providing development opportunities, commitment from leadership to recognize and invest in staff and supporting self-care. Focusing on ensuring internal organizational justice for staff may also facilitate resilience to external challenging environments. Better supporting clinic staff is likely also important for quality services and ensures the full workforce involved in providing family planning care can work at full capacity.

## Significance Statement

*What is already known?*: Several studies have focused on the need for recruitment and training of abortion providers, particularly physicians and residents, as well as challenges with physician burnout, but few studies address the needs of clinic support staff.

*What this study adds?*: The non-clinician staff of family planning and abortion clinics are rarely discussed in conversations about sustaining access to sexual and reproductive health services. Yet, staff are essential components in maintaining accessibility of care and quality of services and staff turnover is often high. This study highlights specific challenges faced by clinic staff and identified potential strategies to address them.

## Introduction

Access to quality family planning (FP) care, including abortion, is critical to sexual and reproductive health (SRH) and maternal health outcomes (Espey et al., 2019). Abortion and FP care workers are particularly vulnerable to high levels of stress and burnout. Nationally, FP clinics are facing shortages of health care providers (NFPRHA, 2010; Ranji et al., 2019). Clinical support staff often have the most interaction with patients and yet are frequently left out of conversations regarding sustaining and expanding access to healthcare. Sustaining these staff are especially important in FP and abortion care, as these fields have been identified as the most challenging to staff in the conservative south and should be a larger part of conversations around sustaining and expanding access to these crucial services (Joffe, 2014; McLemore et al., 2015; Mercier et al., 2016). Individuals frequently choose to receive services at specialized FP clinics^1^ for example, because of access to confidential care, respectful, and knowledgeable staff (Frost et al., 2012b).

The recruitment and retention of clinicians who provide reproductive care, especially those in FP services, are costly tasks (NFPRHA, 2010; Mercier et al., 2016). Physician burnout and turnover also contributes to lower productivity, to diminished quality of care, patient safety, and to a degradation of patient experience (Blue Ridge Group, 2018; Martin et al., 2014). Recently, some studies have examined high rates of turnover and burnout among abortion clinic support staff, noting the need for a formalized support structure to addresses these challenges (Debbink et al., 2016; Janiak et al., 2017). Few published studies, however, have identified the reasons for turnover and strategies for recruitment and retention of these vital staff.

The Southern United States provides a particularly important example, given the often-noted shortages of FP providers, the hostility toward abortion rights, and the stigma around FP in the region (Cheng et al., 2014; Jones & Jerman, 2017; Joyce, 2011; Martin et al., 2014; Nash, 2018). Ensuring access to FP and abortion services in these areas is also important given the context of high rates of maternal morbidity and mortality in these regions (Verma & Shainker, 2020). The number of abortion providers in Southern states, for example, is 45% fewer than in non-Southern states, and the average percentage of women aged 15–44 living in a county without an abortion provider in Southern states is significantly higher than in non-Southern states (56% vs 38%) (Frost, 2016).

This qualitative study sought to better understand factors contributing to the turnover of support staff at FP clinics in seven Southern states. We defined support staff as non-clinician staff working in health services-related roles (e.g., health center assistants, clinic administrators, billing, front desk or call center staff). This qualitative study aimed to identify factors related to turnover as well as creative strategies to better support clinic staff in the FP healthcare environment.

## Methods

### Study Design and Recruitment

This study was part of a larger evaluation of FP programs in the South. The evaluation focused on specialized FP clinics in seven Southern states, which included Alabama, Florida, Georgia, Kentucky, Mississippi, Tennessee, and Louisiana. Staff and leadership from participating organizations were asked to participate in semi-structured in-depth interviews. The study was reviewed and received exempt status from the Emory Institutional Review Board. The reporting of this study follows COREQ criteria (Tong et al., 2007).

With our evaluation partner, our team identified two to three individuals per site who had working knowledge of clinic service delivery. The first and senior authors, female, academics and experienced qualitative researchers, conducted 19 in-person, individual interviews with staff and leadership between January and June 2017. Our sample ranged in age, number of years with the organization, race/ethnicity, and degree of urbanicity. We also ensured diversity in representation of roles within organizations including those in leadership or administrative roles and those involved in direct patient care (health center managers).

Interviews lasted 45–70 min and included a range of topics on providing FP services, including workforce recruitment and retention barriers and facilitators. Interviews were voluntary and conducted in confidential spaces. Prior to the interview, participants were given information about the study and provided their verbal consent. They did not receive an incentive for participation.

### Analysis

Interviews were audio-recorded, professionally transcribed, and analyzed using MaxQDA (VERBI Software, 2018). Thematic analysis was used to identify prominent themes by simultaneously applying deductive codes based on interview guides and inductive codes derived directly from participants’ responses (Braun & Clarke, 2006). The first authors, with the assistance of two research assistants, coded all transcripts and team members met to discuss discrepancies in coding until a consensus was reached. Preliminary results were shared with interviewees and program stakeholders to ensure accuracy. Of the 19 interviews conducted, we included 15 in this analysis as they addressed topics related to clinical workforce.

## Results

The majority (*n* = 10) of the participants were in leadership roles, and five were clinic support staff. Participants discussed the implications of clinic turnover and identified several challenges related to recruitment and retention as well as several strategies with which they had had success (Table 1).

**Table 1:** Frequency of major themes related to recruitment and retention across participants in leadership or clinic staff roles among participants in seven Southern states

Theme	Leadership	Clinic staff	Total
Challenges			
Transparency in decision-making and communication	3	3	6
Conservative context/stigma	3	2	5
Geographically rural locations	2	2	4
Perceived value of support staff	1	2	3
Strategies for success			
Creating advancement opportunities	2	5	7
Prioritize investment in management	4	2	6
Prioritizing staff retention	2	3	5
Self-care strategies	1	1	2

### Implications of Turnover

Respondents emphasized that support staff were essential in maintaining the accessibility and quality of services at their clinics. Respondents reported high turnover staff rates within their organizations ranging from 33% to 100% in the year prior to the interviews. High staff turnover disrupted clinic schedules and flow because the staff that remained had not only to compensate for the work no longer performed by the staff who left and thus were not able to see as many patients.

“[Turnover] impacts the numbers of patients we’re able to schedule and it impacts their wait times while they’re here.” (Tennessee Leadership)

Maintaining a steady schedule was also difficult for these clinics when they were continuously trying to train new support staff.

“I think you have to struggle with quality…if you’re constantly bringing on a new team member that doesn’t have that experience or skillset, yet.” (Kentucky Leadership)

Participants from nearly half of the organizations also noted that turnover and the subsequent time spent re-training wasted financial resources and put additional strain on already tight budgets. Respondents reported that newly trained staff would often leave within a few months, which meant further expense of time, money, and other precious resources.

Participants indicated that high turnover ultimately reduced access to quality care for the patient. When the clinic was not operating with a fully trained staff, the burden was not only on the clinic, but was also on the patient, who had to wait longer for care or receive care from someone being pulled in several different directions.

### Challenges for Retaining Staff

Themes associated with challenges for retaining staff are summarized in Table 2 with exemplar quotes.

**Table 2:** Challenges for family planning support staff recruitment & retention themes and exemplar quotes among participants in seven Southern states

Theme	Quote
Transparency in decision making & communication	“[We need to] get more input from our front-line staff and our clinicians. For a long time, our [organization] has been “okay here’s a problem. We’re going to have four people that sit in the [administrative] office figure out a solution and then we’re going to tell you what that is. And we’ve just not really had the level of engagement and involvement that we have needed from the people that are actually doing the work.” (Louisiana Clinic Staff)
Conservative landscape & stigma	“We’re in a very religious community, very conservative community …Just working for [FP clinic] is challenging because of the stigma associated with us so for lots of reasons we are always understaffed, we are always struggling, we don’t seem to ever be operating at capacity for more than a month at a time.” (Tennessee Leadership)
	“[…] you might worry about your children or your spouse or your future job prospects if [FP center] is on your resume and you intend to continue living in the south. I think people have real fears about being associated with [FP center] professionally, because of the stigma associated with abortion.” (Regional Management)
Geography	“We have to usually send staff [a long distance] for training…And they need that training for a minimum of two weeks […] And then ideally, we would like a preceptor ….to be shadowing that trainee. That hasn’t always been possible. And a lot of our training has been kind of piecemeal, unfortunately… [the clinic] is so far from the locus of the rest of the [organization].” (Florida Clinic Staff)
Perceived value of support staff	“We just don’t pay. I mean [a clinic staff member] can make a lot more money at a hospital down the street than they can with [our FP clinic], so they have to be really connected to the mission.” (Kentucky Clinic Staff)

#### Transparency in Decision-Making and Communication

The most frequently identified challenge was a gap in communication between leadership and clinic staff. Clear communication with staff regarding organizational decision-making suffered because those in leadership roles were frequently dealing with external crises brought on by the difficult social context in which the clinics worked. Participants noted that inconsistent communication with leadership left the clinic staff feeling undervalued, contributing to high turnover.

One respondent noted that when staff were not actively involved in relevant decision-making processes, especially in regard to decisions which directly affected their day-to-day work, frustration built up and ultimately turnover increased. Clinic staff also did not understand why they were not part of this process. Respondents in both staff and leadership positions emphasized the importance of clinic staff knowing how and why decisions were being made by leadership.

#### Conservative Landscape and Stigma

Working in the field of FP and abortion within the context of the conservative South carried stigma. Respondents in both staff and leadership positions emphasized the impact that working in conservative communities had on the staff. For most respondents, the stigma attached to working in FP was associated with providing abortions, which seemed pervasive regardless of whether the clinic was providing abortion care or not. This stigma further contributed to challenges in staff recruitment and retention, and ultimately negatively impacted turnover within the clinic setting.

Some staff worried about their safety coming and going from the clinic and indicated that this feeling of uneasiness in their own community significantly contributed to the retention of existing as well as the recruitment of new staff. For those clinics providing abortion services in conservative areas, the recruitment of staff was even more challenging.

In some instances, the community stigma associated with working in abortion care caused some staff to be “blacklisted” from other potential jobs and roles within the community. Other respondents described incidents in which children of people working with these clinics were also harassed at school and in their neighborhoods.

#### Geography

The remote and rural locations of some clinics also negatively affected staff turnover and retention. Remote sites had small pools of qualified applicants and few resources to provide for professional development.

Some clinics that acted as part of a larger organization, and those located relatively far from headquarters found it hard to stay connected and feel involved. Providing trainings, for example, presented a challenge, as the trainings were scheduled to occur at the headquarters, often a far distance away.

These rural sites often experienced periods without an on-site supervisor. Although leadership from headquarters made efforts to travel to the remote sites, respondents noted that it was difficult for those traveling to connect with staff. It was also difficult to provide guidance remotely or in-person intermittently. This deepened the disconnect between staff at the rural site and the rest of the organization which participants linked to higher turnover.

#### Perceived Value of Support Staff

Another overarching theme was that support staff often felt under-valued. Several respondents noted that staff craved opportunities to be engaged and to move up within the organization but that opportunities were not often offered. A temporary solution was to provide additional training, but many sites did not have a formal, standardized training program. This resulted in inconsistent training experiences and frustration.

Respondents also acknowledged that there was a perceived expectation that clinic staff would work long hours, under highly stressful conditions, and with low pay simply for the sake of the mission. While the mission of their work did motivate staff members, their hard work eventually took a toll, especially when leadership did not acknowledge that the staff were a valued part of the larger organization.

The combination of challenging environments, expectations of “the mission” driving one’s work with a perceived lack of recognition and low pay ultimately generated difficulties in maintaining staff. Although this theme of staff feeling undervalued emerged from interviews with respondents in both leadership and clinic staff positions, it was more prominent in interviews with staff.

### Strategies for Recruitment and Retention

Participants also identified several key strategies for better supporting and retaining FP clinic staff in the South (Table 3). Several of the strategies mirror or engage the challenges identified above.

**Table 3:** Strategies for family planning support staff recruitment & retention themes and exemplar quotes among participants in seven Southern states

Theme	Quote
Create advancement opportunities	“[We would have] local representatives from public affairs who attend health center meetings, who [provide] updates [for] staff, and when possible, … invite staff to attend certain events…and I thought it was very successful because it got health center staff engaged on a whole different level that they don’t get to do in their day-to-day capacity.” (Florida Clinic Staff)
Invest in management	“If you’re not putting those initial roles in a place to be successful, I think it creates problems for those who are hired under that type of leadership.” (Regional Management,)
Prioritize staff retention	“Communicating with everybody and…taking a moment to show that I care about their time and that I understand that they’re gonna be stretched thin and that they’re gonna be working really hard and…making sure they know I appreciate it.” (Kentucky Clinic Staff)
	“Very few leave because they get a better job elsewhere. We conduct stay interviews. […] The overall goal is like ‘if you got offered this same job somewhere else, would you take it?’ And if so, why and what can we do to – so identifying those high performers and having that interview. Instead of an exit interview, having a stay interview; ‘what do we need to do to keep you here?” (Louisiana Clinic Staff)
Invest in self-care strategies for staff	“…At the end of the day our job is to make sure that the patient leaves with a little less burden than when she came in. And when you look […] at energy transfer, energy, you can’t destroy it; it moves. So that burden isn’t destroyed, it’s moved. And we take on that burden. So as her healthcare provider, we take a little of that burden from her or we take all of it or some of it or most of it. And then she leaves, and we hold on to that. And I think that we are doing a disservice to our medical services staff by not giving them the ability to unload that burden before they go home at the end of the day. […]” (Louisiana Clinic Staff)

#### Create Advancement Opportunities

Participants noted that keeping staff challenged was important. For example, identifying where staff could be in charge, or be “the expert” in certain processes in the clinic, such as supervising inventory or maintaining a specialized machine, was a helpful approach. If the clinic participated in training clinical providers, encouraging the clinic staff to assist in that training was also way to keep the staff feeling engaged and valued.

Providing opportunities to network and to make more professional connections locally and nationally was also identified as a strategy. People wanted to know others in similar roles so that they didn’t feel as isolated. Supporting and encouraging clinic staff to work within the advocacy and public affairs realm was also beneficial in that it engaged the staff in a new way and ultimately helped frame their work in a broader context.

While respondents from both the leadership and the clinic staff identified opportunities for career advancement as important, this theme was more frequently discussed by clinic staff with every staff respondent identifying this as a successful strategy for retention.

#### Invest in Management

Effective management within the clinic was key to better support and retention of staff. Effective management meant giving individuals the appropriate skills and tools to succeed, training staff from within, and involving staff in decisions. Respondents felt that it was vital to hire individuals who had demonstrated success in management. For those in management who lacked prior experience, manager specific training and mentoring helped develop skills. Part of ensuring strong management within the clinic included ensuring that managers had time to truly manage the staff and act as a leader. Due to the high turnover of staff, clinic managers often had to act as an additional clinic assistant rather than being able to do their intended work.

Off-site leadership often made decisions related to everyday work at the health center. Involving clinic managers in the decision-making process as a representative for their staff was identified as beneficial and successful.

Additionally, the ability of leadership to seriously solicit and consider feedback from the front-line staff was identified as vital to improving FP clinic staff retention. Nearly all the participants who responded that an investment in management was of key importance were in leadership roles.

#### Prioritize Staff Retention

Respondents observed that while encouraging career development and learning new skills was important, making sure the clinic staff knew they were appreciated was also vital, especially from those in higher leadership positions. Even if no formal staff recognition program existed, respondents emphasized the importance of making sure that staff knew that those in leadership understood and appreciated their work.

One respondent described an innovative and successful strategy for addressing turnover by prioritizing staff retention by conducting “stay interviews” to learn how to better retain staff.

Participants responded that when leadership went above and beyond to recognize staff development, particularly if pay raises for staff were not possible, the results were a happier staff and a noticeable decrease in turnover.

#### Invest in Self-Care Strategies

As noted above, conservative contexts produced challenges to retaining staff, and respondents emphasized that in response to this it was critical to equip staff with skills for self-care. Providing, and encouraging support staff to find, healthy outlets for alleviating stress and getting relief from the weight of their work was particularly important.

Although some respondents recognized that health center managers sometimes made efforts to show their staff appreciation, the expense of these efforts was not covered by their organization. Respondents underscored the benefits for the organization of encouraging and investing in self-care activities for front-line staff, since these staff can often carry the burden and experience incidences of trauma during their day-to-day jobs. Leadership and staff endorsed the importance of providing self-care equally and attributed this to a happier, healthier workforce within the clinic.

## Discussion

This qualitative study found that clinic staff turnover, is especially challenging for specialized FP and abortion clinics and that it can contribute to a poorer experience for patients. We identified several contributing factors to poor recruitment and retention of FP and abortion clinic staff including communication challenges and the need to involve staff in decision making and provide them with opportunities for growth. Similar to other studies of abortion providers, we found that FP staff were subject to stigma as well as high levels of stress, which can contribute to turnover as well as challenges in recruitment (Martin et al., 2014; O’Donnell et al., 2007). Of particular relevance to the South, we found that communication between leadership and staff suffers in challenging environments where leadership must often focus on “putting out fires.” Additionally, clinics that were geographically isolated experienced difficulty retaining staff simply due to the distance and availability of resources to train their staff. Many workforce factors identified are similar to other experiences in resource constrained settings such as community health centers (Rosenblatt et al., 2006).

Participants identified several strategies with which to address these challenges, including the provision of opportunities for growth, investment in management, and finding ways to recognize staff. Given a conservative environment and taxing work, participants emphasized the importance of investing in self-care for staff. Staff and leadership who reported using strategies to support and retain their staff noted that there were improvements in the effectiveness of their work as well as increases in the volume of patients served at their clinic.

**Fig. 1:**
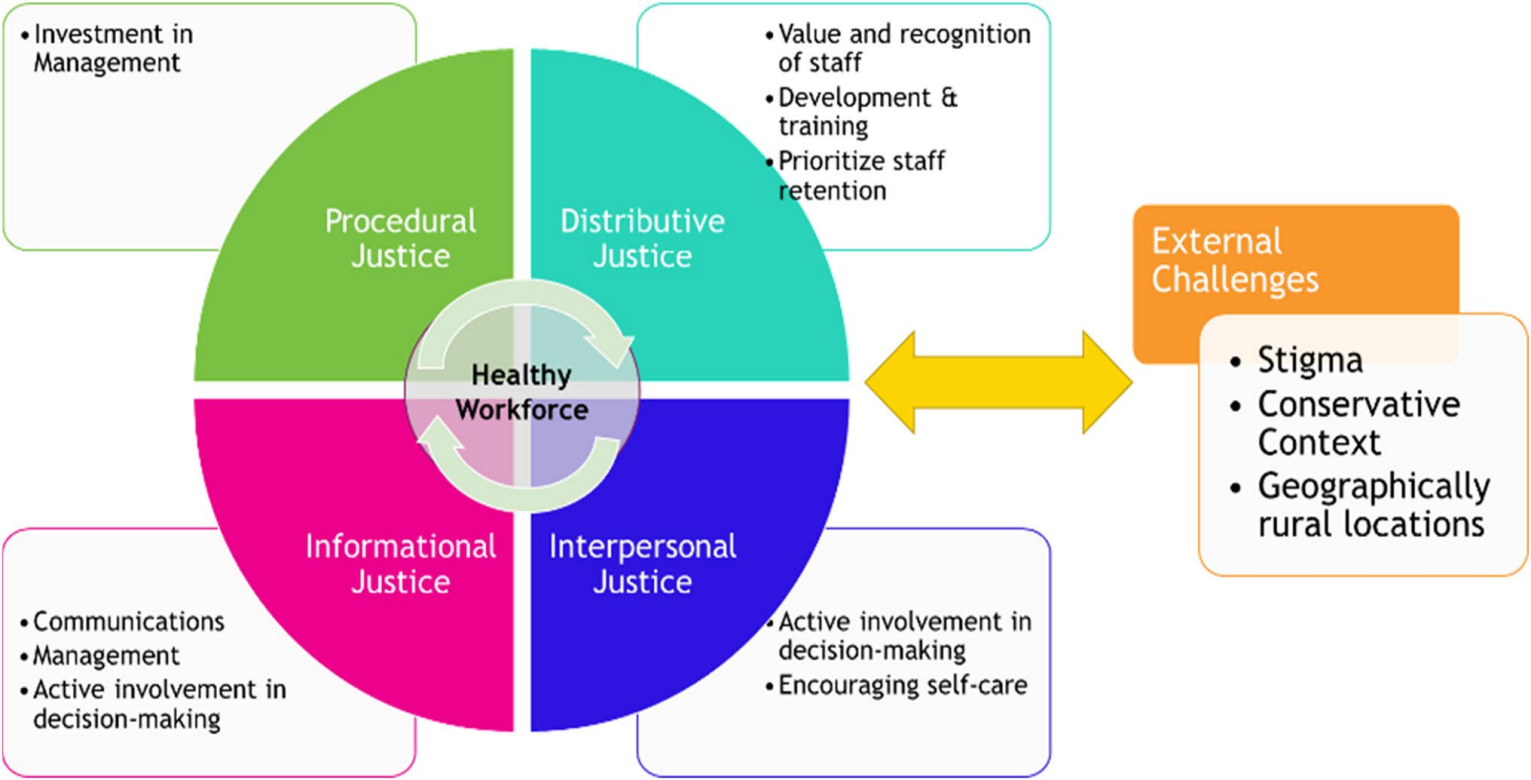
Organizational justice framework for supporting FP & abortion staff, Top

Organizational justice, defined as employees’ subjective perception of workplace procedures, interactions, and outcomes as fair or unfair, provides a helpful lens for understanding many of these themes (Greenberg, 1987). Organizational justice has been previously employed to understand clinical provider burnout and challenges with retention and turnover (Vaamonde et al., 2018). We tie study themes into an organizational justice framework encompassing four major constructs of organizational justice, and we use this frame to show how healthy workforce practices can be promoted within the clinic (Fig. 1). *Procedural justice*, or the perceived fairness of management policies and procedures in determining outcomes and relates to trust, job satisfaction, and perceived organizational commitment. We identified the theme of “investing in management” as fitting within this construct. *Distributive justice* (the perceived fairness of how resources are distributed) encompasses study themes relating to the value and recognition given to staff as well as to provision of opportunities for development and training. Everyone within FP clinic settings is stretched, but support staff in particular may not receive adequate recognition for the intensity of work they perform as others in higher positions. *Informational justice* (the manner in which outcomes and procedures are communicated), encompasses themes of improved communications, management, and involvement in decision-making. Finally, i*nterpersonal justice* (the expectation of respectful treatment) aligns with themes related to recognizing staff and encouraging self-care.

Studies of healthcare workforce have found a combination of procedural and interactive (informational and interpersonal) justice elements to be most important (Chen et al., 2015; Cunningham et al., 2013; Mohamed, 2014). While in our findings, procedural and interactive themes came up frequently, many themes also heavily emphasized *distributive justice* in relation to appropriately valuing and compensating staff. Working for the mission will only take people so far. Thus, it is important for management to ask “are the clinic support staff getting as much back as they are putting into the work?”.

This study addresses an additional component not frequently addressed by organizational justice– that of external challenges and threats (including stigma, conservative contexts, and rural geographical locations), and so we extend this framework as well. We theorize that supporting organizational justice may build resilience to difficult external factors. Further studies are needed to examine the potential for this relationship.

While this study provides important perspectives of both those in leadership roles and those working within clinic settings, a few limitations should be considered. First, this study does not represent all of the perspectives of FP employees. For example, we did not interview staff in each support position within the clinic, including front desk staff. FP clinics with more resources may also be better able to cope with high turnover of clinic staff. Other studies have found that staff in hospitals tend to experience less burn-out than independent clinics (Janiak et al., 2018). This study however provides an important contribution in identifying underlying reasons for turnover and potential strategies for addressing it.

## Conclusion

To date, the majority of literature on FP and abortion care workforce has focused on medical students, providers, and nursing staff (Cheng et al., 2014; Debbink et al., 2016; Janiak et al., 2017; Martin et al., 2014; McLemore et al., 2015; Mercier et al., 2016; NFPRHA, 2010). Support staff are equally as important to the functioning of the clinic, and often don’t have as much agency within clinics. High staff turnover has implications for the quality of care that these clinics are able to provide. Strong support of these staff, especially in the challenging environment of the South, is critical to ensuring access to SRH services. This is particularly important in locations with high rates of maternal morbidity and mortality such as those in the South. Our findings demonstrate the importance of investing in management practices that promote equity and prioritize the retention of support staff, as well as providing opportunities to develop professionally. Clinical staff are part of an essential team, and it is vital to include them in communications and decision making where possible. Examples of this could be all-team debriefings on difficult patients, or problem-solving dyads or triads with a provider or an administrator. Finally, our findings suggest that all staff cannot thrive off the mission alone. In the South, clinics face challenges related to the conservative environment which ultimately leads to staff experiencing burnout and the effects of stigma. Future research is needed to investigate fostering the mental health and well-being of support staff in FP clinics. Given the current shifting and increasingly hostile context for SRH throughout the US, the challenges and strategies this study uncovers may be applicable to understanding workforce retention in challenging contexts outside the South (Aiken & Scott, 2016; Gold & Hasstedt, 2017). FP and abortion services are a critical component of the continuum of reproductive healthcare and ensuring access to these services is important for achieving positive maternal health outcomes. Identifying opportunities for better supporting clinic staff may provide key areas of intervention aimed at ensuring that the whole workforce providing FP care can work to their full capacity.

## Data Availability

Not applicable.

## Footnotes

1 Specialized FP clinics may be distinguished from safety net or primary care focused clinics in that they 1) are not connected with any broader health system, 2) specialize in providing comprehensive family planning and SRH care, including a full range of contraceptive methods and, 3) often have unique financial structures or sources of payment. (Frost et al,2012a).
